# Cephalometric norms for the upper airway of 12-year-old Chinese children

**DOI:** 10.1186/1746-160X-10-38

**Published:** 2014-09-13

**Authors:** Min GU, Colman PJ McGrath, Ricky WK Wong, Urban Hägg, Yanqi Yang

**Affiliations:** 1 Orthodontics, Faculty of Dentistry, the University of Hong Kong, 34 Hospital Road, Hong Kong, SAR, China; 2 Public Health, Faculty of Dentistry, the University of Hong Kong, 34 Hospital Road, Hong Kong, SAR, China; 3 Department of Dentistry and Maxillofacial Surgery Cleft Centre, United Christian Hospital, Hong Kong, SAR, China

**Keywords:** Cephalometry, Chinese, Children, Upper airway

## Abstract

**Objective:**

To establish cephalometric norms for the upper airway of 12-year-old Chinese children, and to assess these norms with regard to gender, age, ethnicity and other craniofacial structures.

**Methods:**

Lateral cephalograms were obtained from a random sample of 425 12-year-old Chinese children (224 boys and 201 girls) to establish the Chinese norms, and from a matched group of 108 12-year-old Caucasian children (61 boys and 47 girls) as an ethnic comparison. Published data on the upper airway norms of Chinese adults were used to make age comparisons. Nine upper airway and 14 craniofacial variables were measured.

**Results:**

Chinese boys tended to have a thicker soft palate (*P* = 0.008), and less depth in the retropalatal (*P* = 0.011), retroglossal (*P* = 0.034) and hypopharyngeal (*P* < 0.001) pharynx than Chinese girls, whereas no gender dimorphism was found in Caucasian children. Ethnic differences were found in the depth of the retroglossal oropharynx in both genders and the position of the hyoid bone in boys. Compared with Chinese adults, the overall size of the upper airway in Chinese children was smaller. The mandibular body length and the craniocervical inclination were found to be statistically significantly, albeit weakly correlated with upper airway variables.

**Conclusions:**

Cephalometric norms for the upper airway of Chinese 12-year-old children were established, indicating gender-specific differences, and some ethnic differences were found in comparison with those of 12-year-old Caucasian children. An association between the mandibular body length and the craniocervical inclination with upper airway variables was also noticeable.

## Introduction

Increasing evidence has shown an association between craniofacial anomalies and/or soft-tissue hypertrophy and pediatric sleep-disordered breathing (SDB) [1-3]. SDB represents a spectrum of breathing disorders during sleep that encompasses a continuum of diagnoses ranging from partial upper airway obstruction (primary snoring and upper airway resistance syndrome) to complete upper airway obstruction (obstructive hypoventilation and obstructive sleep apnea syndrome [OSAS]) [4]. In an updated clinical practice guideline on childhood OSAS from the American Academy of Pediatrics, SDB was defined simply as OSAS with additional snoring [5]. The prevalence of children with OSAS ranges from 1 to 5%, and the reported prevalence of habitual snoring varies widely from 1.5 to 27.6% [5]. If untreated, pediatric SDB may result in serious problems such as a failure to thrive, neurocognitive deficits, behavioral abnormalities and cardiovascular changes [6]. The common modalities of treatment for pediatric SDB include adenotonsillectomy, continuous positive airway pressure (CPAP), oral appliances and pharmacologic treatment [7].

The soft-tissue morphology of the upper airway that may related to pediatric SDB includes narrowed pharyngeal airways, and larger adenoids, tonsils and soft palates [8-10]; the related hard-tissue morphology includes an increased intermaxillary angle, a retrognathic mandible [11,12], an increased mandibular angle, a longer lower anterior facial height [13,14], narrow dental arches and deep palatal height [15,16], and an inferiorly positioned hyoid bone [17,18].

Both upper airway and craniofacial structures can be observed in the same lateral cephalograms, and lateral cephalometric radiography was therefore considered to be a useful screening tool to assess upper airway structures, and identify subjects at risk of SDB [19,20]; its validity has been tested using three-dimensional computed tomography (CT) and magnetic resonance imaging (MRI) [21,22].

To assess the upper airway structures of individual patients from lateral cephalograms, reference norms are required. However, there is limited data on cephalometric norms of upper airway. McNamara [23] provided reference values for the upper pharynx and lower pharynx of Caucasian adults, whereas Samman et al. [24] presented cephalometric norms for the upper airway of Chinese adults. Until now, no cephalometric norms for upper airway of Chinese children has been established. Consequently, the objective of this retrospective study was to obtain upper airway norms for Chinese children, and study the association between airway dimensions and craniofacial structures.

## Materials and methods

### Subjects

The materials used were lateral cephalograms obtained from 12-year-old children during an oral health survey performed in 1984-85 at the Department of Orthodontics and Paediatric Dentistry of the University of Hong Kong [25]. In the survey, approximately 600 Chinese school children were selected by a partially stratified random sampling from 10 schools in Hong Kong, and approximately 100 Caucasian school children, whose parents originated from the United Kingdom, were chosen from two expatriate schools in Hong Kong [26]. Lateral cephalometric radiographs were taken as part of a comprehensive oral/dental/facial examination. The lateral cephalograms used in this study were selected according to the following criteria: 1, children aged 12.0 -13.0 years; 2, who had not received or were not receiving ongoing orthodontic treatment; and 3, the upper airway structures were clear and no swallow action was detected in the cephalograms. After exclusions, lateral cephalograms from 224 male and 201 female 12-year-old Chinese school children, and 61 male and 47 female 12-year-old Caucasian school children were studied (Table 1).

**Table 1:** Demographic data of the study samples

		**Age range**			**Range**	
	**n**	**Mean**	**SD**	**Median**	**Minium**	**Maxium**
Chinese children						
Male	224	12.5	0.3	12.5	12.0	12.9
Female	201	12.5	0.3	12.5	12.0	12.9
Difference^a^		0.0				
Chinese adults						
Male	29	-	-	-	18.0	35.0
Feame	45	-	-	-	16.0	42.0
Difference^b^		-				
Caucasian						
Male	61	12.5	0.2	12.5	12.0	12.9
Female	47	12.4	0.3	12.5	12.0	12.9
Difference^a^		0.1				

^a^None of the differences between the samples were statistically significant.

^b^Statistically significance could not be calculated.

### Ethics issue

The present study was approved by the Institutional Review Board of the University of Hong Kong/Hospital Authority Hong Kong West Cluster (IRB Reference Number: UW 12-405).

### Radiographic technique

One X-ray machine (GE1000, General Electric, Milwaukee, Wits) was used to obtain all lateral cephalograms. The magnification was 8.8% for the midsaggital structures. The lateral cephalograms were obtained during a natural head posture, in which the subjects looked at the reflection of their eyes in a mirror placed 200 cm in front of them after first tilting their head forward and backward with decreasing amplitude until a comfortable position of natural balance was found [25].

### Cephalometric analysis

The sample of Chinese children was used to establish cephalometric norms for the upper airway of Chinese boys and girls, respectively. The sample of Caucasian children was used for an ethnic comparison. Published data on the upper airway norms of Chinese adults were used for an age comparison [24]. The demography of all samples is presented in Table 1.

**Figure 1:**
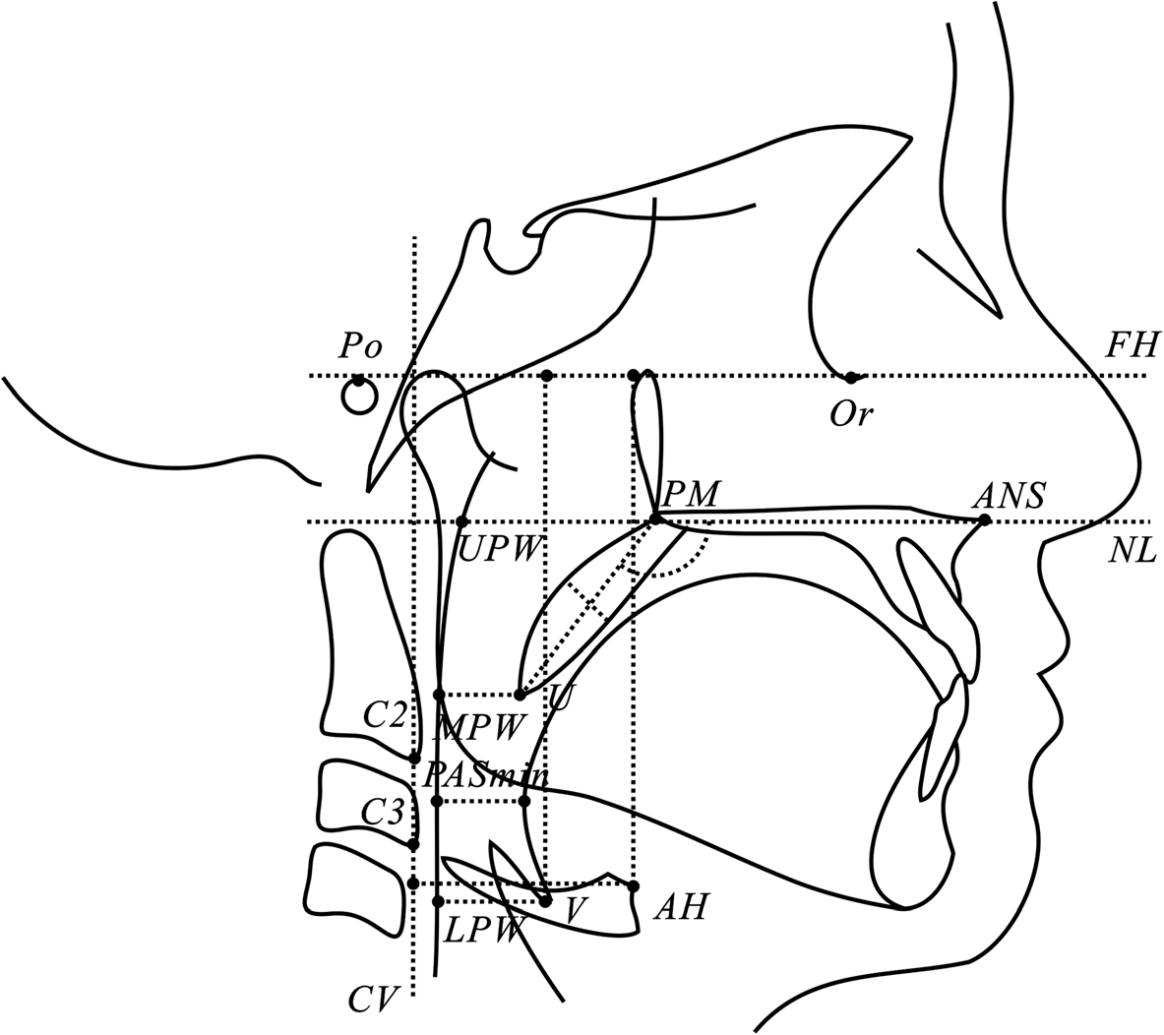
Landmarks and measurements of the upper airway.

**Figure 2:**
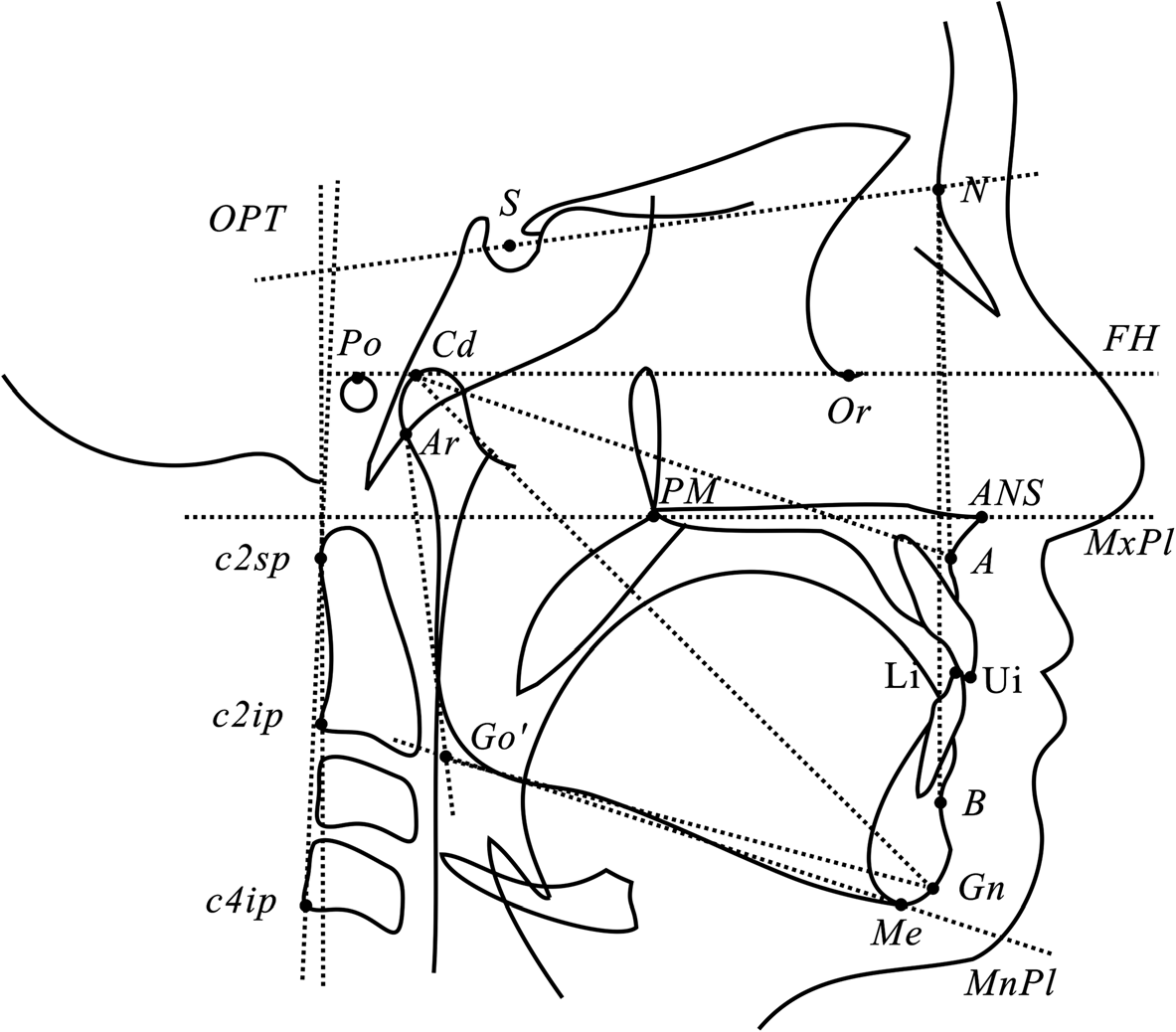
Landmarks and measurements of the craniofacial structures.

The landmarks and reference lines of the upper airway are shown in the Table 2 and Figure 1. The variables for upper airway measurements included eight linear variables and one angular variable (three variables for the soft palate, four variables for the upper airway depth and two variables for the position of the hyoid bone), which were selected from a previous study on Chinese adults [24]. The analysis of the craniofacial morphology included six linear and eight angular conventional measurements (Table 3 and Figure 2) [11,27]. The analysis was carried out using CASSOS software (Soft Enable Technology Limited, Hong Kong, China). All of the linear measurements were corrected according to the magnification.

**Table 2:** Cephalometric landmarks and measurements of the upper airway

**Variables**	**Definition**
*Landmarks*	
Po	Porion, the mid point of the line connecting the most superior point of the external auditory canal on both sides
Or	Orbitale, the lowest point on the average of the left and right inferior borders of the bony orbit
ANS	Anterior nasal spine, the tip of the median, sharp bony process of the maxilla
PM	Pterygo-maxillare, the point at the junction fo the pterygo-maxilla and the posterior nasal spine
U	Uvula, the tip of the uvula
UPW	Upper pharyngeal wall, point of intersection of the line NL to the posterior pharyngeal wall
MPW	Middle pharyngeal wall, intersection of the perpendicular line from U to the posterior pharyngeal wall
LPW	Lower pharyngeal wall, intersection of the perpendicular line from V to the posterior pharyngeal wall
V	Vallecula, the intersection of the epiglottis and the base of the tongue
AH	Anterior hyoid, the most anterior and superior point on the body of the hyoid bone
C2	2nd cervical vertebrae, the point at the most anterior-inferior position on the second cervical vertebrae
C3	3rd cervical vertebrae, the point at the most anterior-inferior position on the third cervical vertebrae
FH	Frankort horizontal plane, line joining the Or to the Po
NL	Nasal line, line joining the ANS and Pm
CV	Cervical vertebrae, the line joining the C2 and C3
*Measurements*	
PM-U (mm)	Length of soft palate, distance from PM to U
SPT (mm)	Soft palate thickness, represents the maximal thickness of the soft palate measured perpendicular to PM-U line
NL/PM-U (°)	Inclincation of the long axis of the soft palate relative to the nasal line
PM-UPW (mm)	Depth of the nasopharyngeal airway space from PM to UPW
U-MPW (mm)	Depth of the oropharyngeal airway space from U to MPW
PASmin (mm)	The shortest distance between the base of the tongue and the posterior pharyngeal wall, the narrowest sagittal airway space
V-LPW (mm)	Depth of the hypopharyngeal airway space from V to LPW
AH-FH (mm)	Position of the hyoid bone in vertical plane, from AH perpendicular to FH
AH-CV (mm)	Position of the hyoid bone in horizontal plane, from AH to CV and parallel to FH

**Table 3:** Cephalometric landmarks and measurements of the craniofacial structures

**Variables**	**Definition**
*Landmarks*	
S	Center of the sella turcica
N	Nasion, the deepest point in the concavity of nasofrontal suture
A	A point, the deepest point in the concavity of the anterior maxilla between the anterior nasal spine and the alveolar crest
Ui	Upper incisor tip
Li	Lower incisor tip
B	B point, the deepest point in the concavity of the anterior mandible between the alveolar crest and the pogonion
Gn	Gnathion, the most anteroinferior point on the bony chin
Me	Mention, the most inferior point on the body chin
Go’	Gonion’ point, the intersection of the tangents of inferior and posterior borders of the mandible
Ar	The intersection of the posterior border of the ramus with inferior surface of the cranial base
Cd	Condylion, the most posterosuperior point of the condylar head
c2sp	The most superior posterior point of the second cervical vertebra
c2ip	The most inferior posterior point of the second cervical vertebra
c4ip	The most inferior posterior point of the fourth cervical vertebra
MxPl	Maxillary plance, equal to NL (ANS-PM)
MnPl	Mandibular plane, line joining Me and Go
OPT	Odontoid process tangent, line joining c2sp and c2ip
*Measurements*	
SNA (°)	The angle between the S-N line and the N-A line
SNB (°)	The angle between the S-N line and the N-B line
ANB (°)	The angle between the N-A line and the N-B line
MnPl/SN (°)	Mandibular plane angle, the angle between the MnPl and the S-N line
MxPl/MnPl (°)	Maxillomandibular plane angle, the angle between the MxPl and the MnPl
MxMn-DF (mm)	Masiilomandibular difference, the length of Cd-Gn minus the length of Co-A
TAFH (mm)	The distance from N to Me
TPFH (mm)	The distance from S to Go
Y axis/FH (°)	The angle between S-Gn line and FH plane
Overjet (mm)	The distance between Ui and Li, parallel to the FH plane
Overbite (mm)	The distance between Ui and Li, perpendicular to the FH plane
OPT-SN (°)	The angle between the OPT and the S-N line
c2sp-c4ip-SN (°)	The angle between the c2sp-c4ip line and the S-N line
Body length (mm)	The distance between Go’-Me

### Method error

One examiner (MG) carried out all of the measurements. During the pilot study, the measurements of 10 randomly selected cephalograms were calibrated by another examiner (YQY), and the method error was calculated by Dahlberg’s formula [28], $\mathrm{ME}=\sqrt{\sum d^{2}/2n}$, which is the repeated measurements of 30 randomly selected cephalograms on separated occasions at a 2-week interval. Σd is the difference between two measurements of a pair, and n is the number of double measurements. The method errors for the linear and angular measurements were not statistically significant and did not exceed 1 mm and 1°, respectively.

### Statistical analysis

The measurements of the upper airway are presented as the mean, median, standard deviation, range and 95% confidence interval. The Mann-Whitney *U*-test was used to calculate the gender, age and ethnic differences, and the levels of statistical significance were *P* < 0.05, *P* < 0.01, and *P* < 0.001. The association between the upper airway and the craniofacial structures was analyzed using Spearman rank correlation, and the statistical significance was set at levels of *P* < 0.05 and *P* < 0.01. Statistical analyses were performed using SPSS software (IBM SPSS Statistics 20, IBM Corp.).

## Results

### Gender differences

In Chinese children, boys had smaller values than girls for almost all of the variables, except for the soft palate thickness (Table 4). The gender differences were statistically significant for soft palate thickness (*P* = 0.008), the depth of the retropalatal (*P* = 0.011) and retroglossal (*P* = 0.034) pharynx, and for the depth of the hypopharynx (*P <* 0.001). No significant gender difference was observed in the Caucasian sample (Table 5).

**Table 4:** Cephalometric norms of upper airway in Chinese subjects and gender differences

**Variables**		**Boys**							**Girls**							**Gender difference**	**P value**	
					**Range**		**95% CI**					**Range**		**95% CI**				
		**Mean**	**SD**	**Median**	**Min**	**Max**	**Lower**	**Upper**	**Mean**	**SD**	**Median**	**Min**	**Max**	**Lower**	**Upper**			
*Soft palate*																		
PM-U	Length of the soft palate	29.6	3.4	29.9	21.0	38.0	29.2	30.1	29.3	3.4	29.1	22.0	45.3	28.9	29.8	0.3	0.271	
SPT	Soft palate thickness	8.3	1.2	8.3	4.1	11.9	8.2	8.5	8.0	1.4	7.9	2.8	12.7	7.8	8.2	0.3	0.008	**
NL/PM-U	Inclination of the long axis of the soft palate relative to the nasal line	125.9	6.1	125.5	109.1	144.2	125.1	126.7	126.9	5.4	127.1	112.9	144.8	126.2	127.7	-1.0	0.058	
*Depth of upper airway*																		
PM-UPW	Depth of the nasopharyngeal airway space	21.0	3.5	21.2	4.7	28.4	20.5	21.4	20.9	3.5	20.9	11.1	28.4	20.4	21.4	0.1	0.553	
U-MPW	Depth of the retropalatal pharyngeal airway space	8.7	2.2	8.6	3.4	14.8	8.4	9.0	9.2	2.4	9.2	3.5	16.0	8.9	9.6	-0.6	0.011	*
V-LPW	Depth of the hypopharyngeal airway space	13.4	3.0	13.9	5.0	19.9	13.0	13.8	14.5	3.1	14.7	5.2	23.2	14.1	15.0	-1.1	<0.001	***
PASmin	Depth of the retroglossal pharyngeal airway space	8.4	2.5	8.4	2.3	14.3	8.1	8.8	9.0	2.7	8.8	2.8	16.5	8.7	9.4	-0.6	0.034	*
*Position of the hyoid bone*																		
AH-FH	Positon of the hyoid bone in the vertical plance	75.2	5.9	75.2	60.8	95.3	74.3	75.9	74.1	6.0	73.9	58.9	99.1	73.2	74.9	1.1	0.04	
AH-CV	Positon of the hyoid bone in the horizontal plance	30.1	3.4	30.1	21.1	37.9	29.6	30.5	30.2	3.0	30.4	22.9	41.1	29.8	30.7	-0.2	0.775	

*p < 0.05; **p < 0.01; ***p < 0.001.

**Table 5:** Cephalometric values of upper airway in Caucasian subjects and gender differences

**Variables**		**Boys**							**Girls**							**Gender difference**	**P value**
					**Range**		**95% CI**					**Range**		**95% CI**			
		**Mean**	**SD**	**Median**	**Min**	**Max**	**Lower**	**Upper**	**Mean**	**SD**	**Median**	**Min**	**Max**	**Lower**	**Upper**		
*Soft palate*																	
PM-U	Length of the soft palate	29.9	3.9	29.7	20.2	42.6	28.8	30.9	29.4	3.3	29.2	23.5	37.9	28.4	30.4	0.5	0.377
SPT	Soft palate thickness	8.3	1.3	8.4	5.2	11.8	8.0	8.7	7.9	1.1	7.9	5.8	10.8	7.6	8.2	0.4	0.055
NL/PM-U	Inclination of the long axis of the soft palate relative to the nasal line	124.6	6.3	125.0	111.0	140.0	123.0	126.2	126.8	6.8	125.0	113.0	147.0	124.8	128.8	-2.2	0.296
*Depth of upper airway*																	
PM-UPW	Depth of the nasopharyngeal airway space	21.0	3.6	21.6	9.9	27.3	20.1	21.9	21.1	4.1	21.0	8.2	31.7	19.9	22.3	-0.1	1.000
U-MPW	Depth of the retropalatal pharyngeal airway space	9.1	2.4	9.1	3.1	13.8	8.5	9.7	9.2	2.3	8.8	4.3	14.2	8.5	9.8	-0.1	0.889
V-LPW	Depth of the hypopharyngeal airway space	13.1	2.5	13.5	8.0	19.7	12.5	13.8	13.1	3.4	13.6	7.4	21.0	12.2	14.1	0.0	0.973
PASmin	Depth of the retroglossal pharyngeal airway space	7.5	2.8	7.4	2.8	13.8	6.8	8.2	7.9	2.8	7.3	4.1	14.7	7.1	8.7	-0.4	0.562
*Position of the hyoid bone*																	
AH-FH	Positon of the hyoid bone in the vertical plance	73.1	5.4	72.8	62.6	84.2	71.7	74.4	72.8	5.2	72.2	60.1	85.9	71.3	74.3	0.3	0.633
AH-CV	Positon of the hyoid bone in the horizontal plance	28.6	2.9	28.8	22.0	35.5	27.8	29.3	29.4	3.0	29.4	24.2	35.6	28.5	30.3	-0.8	0.208

### Age differences

Chinese adults had statistically significant larger values than Chinese children in both genders for all of the variables except for the inclination of the soft palate in males, and the differences were larger in males (Table 6).

**Table 6:** Cephalometric values of the upper airway for interethnic differences (Chinese versus Caucasian)

**Variables**		**Boys**			**Girls**		
		**Mean**	**P value**		**Mean**	**P value**	
*Soft palate*							
PM-U	Length of the soft palate	-0.2	0.681		-0.1	0.954	
SPT	Soft palate thickness	0.0	0.827		0.0	0.714	
NL/PM-U	Inclination of the long axis of the soft palate relative to the nasal line	1.3	0.252		0.2	0.456	
*Depth of upper airway*							
PM-UPW	Depth of the nasopharyngeal airway space	0.0	0.914		-0.2	0.738	
U-MPW	Depth of the retropalatal pharyngeal airway space	-0.4	0.146		0.1	0.724	
V-LPW	Depth of the hypopharyngeal airway space	0.3	0.314		1.4	0.01	**
PASmin	Depth of the retroglossal pharyngeal airway space	0.9	0.019	*	1.1	0.005	**
*Position of the hyoid bone*							
AH-FH	Positon of the hyoid bone in the vertical plance	2.1	0.014	*	1.3	0.114	
AH-CV	Positon of the hyoid bone in the horizontal plance	1.5	0.001	**	0.9	0.074	

*p < 0.05; **p < 0.01; ***p < 0.001.

### Ethnic differences

The 12-year-old Chinese children had larger values for all variables (Table 7). In males, the statistically significant differences were found in the shortest distance of the oropharynx (*P* = 0.019), and the position of the hyoid bone in the vertical and horizontal planes (*P* = 0.014 and *P* = 0.001); and in females, statistically significant differences were found in the depth of the retroglossal pharynx (*P* = 0.005) and that of the hypopharynx (*P* = 0.01).

**Table 7:** Cephalometric values of the upper airway for age differences (adults versus children)

**Variables**		**Males**			**Females**		
		**Mean**	**P value**		**Mean**	**P value**	
*Soft palate*							
PM-U	Length of the soft palate	4.7	<0.001	***	1.3	0.009	**
SPT	Soft palate thickness	1.8	<0.001	***	0.9	<0.001	***
NL/PM-U	Inclination of the long axis of the soft palate relative to the nasal line	1.4	0.119		1.4	0.039	*
*Depth of upper airway*							
PM-UPW	Depth of the nasopharyngeal airway space	4.9	<0.001	***	3.2	<0.001	***
U-MPW	Depth of the retropalatal pharyngeal airway space	1.2	0.017	*	0.7	0.027	*
V-LPW	Depth of the hypopharyngeal airway space	5.3	<0.001	***	2.0	<0.001	***
PASmin	Depth of the retroglossal pharyngeal airway space	1.7	0.002	**	1.0	0.008	**
*Position of the hyoid bone*							
AH-FH	Positon of the hyoid bone in the vertical plance	17.2	<0.001	***	4.4	<0.001	***
AH-CV	Positon of the hyoid bone in the horizontal plance	6.3	<0.001	***	1.0	0.009	**

*p < 0.05; **p < 0.01; ***p < 0.001.

### Correlations between the upper airway and other craniofacial structures

The correlations between the upper airway and craniofacial structures were weak, and no correlation coefficient exceeded 0.5. In Chinese children, the variables of the body length of the mandible and the craniocervical inclination were significantly associated with most of the upper airway variables. The vertical and horizontal position of the hyoid bone was significantly associated with anterior and posterior facial height (Table 8). The results for the Caucasian children were generally similar to those of the Chinese children (Table 9).

**Table 8:** Results of Spearman’s correlation analysis for Chinese samples

		**Soft palate**						**Depth of the upper airway**								**Position of the hyoid bone**			
		**PM-U**		**SPT**		**NL/PM-U**		**PM-UPW**		**U-MPW**		**V-LPW**		**PASmin**		**AH-FH**		**AH-CV**	
SNA (°)	Coefficient	-0.044		0.061		-0.167	**	0.047		0.085		0.042		0.042		0.011		0.064	
	*P* value	0.367		0.211		0.001		0.337		0.082		0.389		0.392		0.826		0.185	
SNB (°)	Coefficient	-0.104	*	0.066		-0.210	**	0.038		0.156	**	0.101	*	0.123	*	-0.019		0.138	**
	*P* value	0.032		0.173		0.000		0.439		0.001		0.037		0.011		0.703		0.004	
ANB (°)	Coefficient	0.124	*	-0.015		0.095		0.026		-0.133	**	-0.083		-0.135	**	0.045		-0.099	*
	*P* value	0.010		0.753		0.049		0.595		0.006		0.089		0.005		0.358		0.041	
MnPl/SN (°)	Coefficient	0.000		-0.046		0.044		-0.111	*	-0.115	*	-0.099	*	-0.068		0.000		-0.121	**
	*P* value	0.997		0.349		0.368		0.022		0.018		0.042		0.164		0.997		0.012	
MxPl/MnPl (°)	Coefficient	-0.088		-0.102	*	0.179	**	-0.023		-0.070		-0.039		-0.025		0.061		-0.099	*
	*P* value	0.071		0.035		0.000		0.632		0.153		0.428		0.603		0.212		0.042	
MxMn-DF (mm)	Coefficient	0.019		0.174	**	-0.191	**	-0.056		0.110	*	0.154	**	0.089		0.263	**	0.251	**
	*P* value	0.701		0.000		0.000		0.252		0.023		0.001		0.067		0.000		0.000	
TAFH (mm)	Coefficient	0.237	**	0.208	**	-0.018		0.112	*	0.062		0.126	*	0.042		0.489	**	0.304	**
	*P* value	0.000		0.000		0.718		0.021		0.203		0.009		0.383		0.000		0.000	
TPFH (mm)	Coefficient	0.201	**	0.210	**	-0.080		0.138	**	0.138	**	0.198	**	0.068		0.417	**	0.389	**
	*P* value	0.000		0.000		0.100		0.004		0.004		0.000		0.161		0.000		0.000	
Y axis/FH (°)	Coefficient	0.122	*	-0.083		0.065		-0.013		-0.159	**	-0.057		-0.141	**	0.139	**	-0.058	
	*P* value	0.012		0.086		0.184		0.789		0.001		0.245		0.004		0.004		0.230	
Overjet (mm)	Coefficient	0.084		-0.034		0.090		-0.001		-0.180	**	-0.081		-0.232	**	0.181	**	-0.089	*
	*P* value	0.085		0.487		0.062		0.990		0.000		0.095		0.000		0.000		0.068	
Overbite (mm)	Coefficient	0.135	**	-0.048		0.023		0.019		-0.134	**	-0.044		-0.150	**	0.080		-0.126	**
	*P* value	0.005		0.324		0.637		0.690		0.006		0.369		0.002		0.100		0.009	
OPT-SN (°)	Coefficient	0.179	**	-0.053		0.336	**	-0.089		-0.017		0.299	**	0.212	**	0.142	**	0.335	**
	*P* value	0.000		0.278		0.000		0.066		0.726		0.000		0.000		0.003		0.000	
c2sp-c4ip-SN (°)	Coefficient	0.195	**	-0.055		0.316	**	-0.103		-0.043		0.289	**	0.169	**	0.171	**	0.278	**
	*P* value	0.000		0.257		0.000		0.033		0.375		0.000		0.000		0.000		0.000	
Body length (mm)	Coefficient	0.051		0.158	**	-0.024		0.166	**	0.204	**	0.206	**	0.224	**	0.290	**	0.302	**
	*P* value	0.296		0.001		0.627		0.001		0.000		0.000		0.000		0.000		0.000	

*Correlation is significant at the 0.05 level.

**Correlation is significant at the 0.01 level.

**Table 9:** Results of Spearman’s correlation analysis for Caucasian samples

**Variables**		**Soft palate**						**Depth of the upper airway**							**Position of the hyoid bone**			
		**PM-U**		**SPT**		**NL/PM-U**		**PM-UPW**		**U-MPW**		**V-LPW**		**PASmin**	**AH-FH**		**AH-CV**	
SNA (°)	Coefficient	-0.010		0.111		0.001		0.178		-0.088		0.040		-0.075	-0.068		0.033	
	*P* value	0.919		0.252		0.991		0.066		0.365		0.684		0.439	0.484		0.738	
SNB (°)	Coefficient	-0.064		0.223	*	-0.106		0.119		-0.098		-0.009		-0.099	-0.032		0.081	
	*P* value	0.508		0.021		0.276		0.221		0.311		0.925		0.308	0.742		0.404	
ANB (°)	Coefficient	0.135		-0.134		0.252	**	0.200	*	-0.069		0.064		-0.005	-0.093		-0.029	
	*P* value	0.162		0.166		0.008		0.038		0.476		0.510		0.958	0.337		0.769	
MnPl/SN (°)	Coefficient	0.026		-0.057		0.103		-0.137		0.145		-0.027		0.065	0.117		-0.043	
	*P* value	0.788		0.557		0.291		0.157		0.133		0.781		0.506	0.229		0.657	
MxPl/MnPl (°)	Coefficient	-0.022		-0.105		0.236	*	0.019		0.104		-0.055		-0.064	0.204	*	-0.100	
	*P* value	0.824		0.281		0.014		0.842		0.285		0.573		0.510	0.034		0.302	
MxMn-DF (mm)	Coefficient	0.050		0.274	**	-0.079		-0.065		0.094		-0.052		0.023	0.423	**	0.153	
	*P* value	0.609		0.004		0.417		0.502		0.332		0.592		0.813	0.000		0.113	
TAFH (mm)	Coefficient	0.240	*	0.180		0.120		0.041		0.086		0.217	*	0.188	0.444	**	0.302	**
	*P* value	0.012		0.063		0.215		0.673		0.373		0.024		0.051	0.000		0.001	
TPFH (mm)	Coefficient	0.257	**	0.269	**	-0.082		0.062		-0.081		0.271	**	0.152	0.348	**	0.291	**
	*P* value	0.007		0.005		0.397		0.521		0.402		0.005		0.117	0.000		0.002	
Y axis/FH (°)	Coefficient	0.188		-0.139		0.003		-0.141		-0.115		0.083		-0.030	0.178		-0.079	
	*P* value	0.051		0.153		0.978		0.147		0.236		0.395		0.757	0.065		0.415	
Overjet (mm)	Coefficient	-0.053		-0.199	*	0.140		0.037		-0.057		-0.050		-0.165	0.096		0.033	
	*P* value	0.583		0.039		0.150		0.701		0.560		0.608		0.087	0.322		0.736	
Overbite (mm)	Coefficient	0.063		-0.034		0.117		0.120		-0.154		-0.076		-0.206	-0.064		-0.150	
	*P* value	0.519		0.729		0.229		0.215		0.112		0.436		0.033	0.510		0.120	
OPT-SN (°)	Coefficient	0.199	*	-0.239	*	0.417	**	0.038		0.302	**	0.353	**	0.404	0.201	*	0.326	**
	*P* value	0.039		0.013		0.000		0.693		0.001		0.000		0.000	0.037		0.001	
c2sp-c4ip-SN (°)	Coefficient	0.213	*	-0.209	*	0.376	**	0.013		0.253	**	0.336	**	0.360	0.202	*	0.278	**
	*P* value	0.027		0.030		0.000		0.892		0.008		0.000		0.000	0.036		0.004	
Body length (mm)	Coefficient	-0.079		0.073		0.206	*	0.318	**	0.280	**	0.136		0.212	0.210	*	0.235	*
	*P* value	0.414		0.451		0.033		0.001		0.003		0.159		0.028	0.029		0.014	

*Correlation is significant at the 0.05 level.

**Correlation is significant at the 0.01 level.

## Discussion

### The rationale for the selection of the upper airway variables

In a lateral cephalogram, the observable SDB-related upper airway structures include: the pharynx, the adenoid, the soft palate, the tonsil, the tongue, and the hyoid bone. The pharynx can be divided anatomically into three parts: the nasopharyx, the oropharynx, and the hypopharynx. The oropharynx can be subdivided into the retropalatal and retroglossal pharynxes [29]. In the present study, we therefore selected four variables to represent four parts of the pharynx. The pharyngeal lymphoid tissues, such as the adenoid and tonsils, were not measured because they begin to atrophy from their maximal size during the pre-pubertal years [30]. No tongue measurement was adopted in the present study because the tongue contour was not clear in the cephalograms without barium sulfate paste [31].

### The influence of gender on the dimensions of upper airway

In the present study it was found that Chinese boys had a thicker soft palate and less depth in the retropalatal, retroglossal and hypopharyngeal regions of the upper airway. It has suggested that these characteristics were related to pediatric SDB [8,10], which may explain why Hong Kong boys had a higher prevalence of OSAS than Hong Kong girls (5.8% versus 3.8%) [32]. In the present there was no difference in the upper airway dimensions of Caucasian boys and girls. Interestingly, no gender difference has been reported in the prevalence of OSAS in Caucasian children by most of studies [5]. Therefore, the upper airway dimensions may be a crucial risk factor of pediatric SDB. However, this seems not to be valid in adults, because men generally have a larger size of pharyngeal lumen than women, but have a higher prevalence of SDB; Subsequently, other factors such as differences in hormones, chemosensitivity and tissue properties may be more important in adult SDB than airway dimensions [29].

### The influence of age on the dimensions of the upper airway

In the present study, the 12-year-old Chinese children, especially the boys, were found to have much growth potential in all of the upper airway structures from childhood to adulthood. The soft palate tended to increase in length, thickness, and inclination, the depth of the pharynx increased at all of the levels, and the hyoid bone moved anteriorly and inferiorly. Chinese boys had more prominent upper airway changes than Chinese girls. In 12-year-olds, Chinese boys had less depth of the pharynx, but they had a larger pharynx in adulthood, showing that Chinese boys have a later spurt in growth compared with Chinese girls. This phenomenon is corresponding to the sexual dimorphism in craniofacial growth [33]. The similar finding was also reported by other lateral cephalometric analysis or 3-dimentional CT research [34,35]. In addition, Taylor et al. [36] reported that the growth of the oropharynx has two periods of accelerated change (6-9 years and 12-15 years) and two periods of quiescence (9-12 years and 15-18 years) for the growth of oropharynx, but Mislik et al. [37] found there was no radical change in the retropalatal and retroglossal oropharynx from 6 to 17 years of age, and considered that the upper airway dimensions were formed and matured during the early periods of growth. Because the chronological regularity of growth of Chinese children is still unclear, we consider that the norms obtained from 12-year-old Chinese children are only relevant to the age around 12-year-old. Wu et al. [38] used the same sample of 12-year-old Chinese children data to get the Chinese norms of McNamara’s analysis and suggested that the norms were suitable for around 10-14 years age. Whether the age range of 10-14 years old is also suitable for upper airway needs further investigation.

### The influence of ethnicity on the dimensions of the upper airway

The prevalence of SDB has been reported to show ethnic/racial differences. African-Americans were considered to have a higher incidence of OSAS than Caucasians [39], and Indians were reported to have a greater risk of SDB than Chinese [40]. No published research has compared the prevalence of pediatric SDB between Chinese and Caucasian children. The present study found the ethnic differences in the upper airways in 12-year-old Caucasian and Chinese children. Both genders of Chinese children had a larger depth of retroglossal oropharynx than their Caucasian counterparts, and Chinese boys had a more anterior and inferior position of the hyoid bone. It is impossible to predict the difference in the risk of SDB between Chinese or Caucasian children based on these findings, because the larger size of pharynx is considered an advantage for airway patency but the anterior and inferior position of the hyoid bone is a disadvantage [17].

### The associations between the upper airway and other craniofacial structures

The present study found that the associations between the craniofacial structures and the upper airway were generally weak, but the mandibular length and craniocervical inclination were found to have more prominent associations with most of the upper airway variables than the other craniofacial structures in both Chinese and Caucasian ethnicities. Ozdemir et al. [41] also reported the a positive association between mandibular body length (GnGo) and minimal posterior airway space (MPAS). The finding seems to indicate that skeletal Class II malocclusion is a risk factor for pediatric SDB. The influence of craniocervical inclination on the dimensions of upper airway has been reported in a number of studies [42-44]. An increase in the craniocervical extension tends to increase the depth of the pharynx, and it was reported to be one of the craniofacial characteristics of children with SDB [19].

### The limitations of the present study

Firstly, because the samples were not from a study that focused on the upper airway, no history-related investigation of the upper airway was made, such as diagnosis of SDB, snoring, enlarged tonsils, or history of adenotonsillectomy, etc. Secondly, the original lateral radiograph technique was not adopted especially for the upper airway, i.e., the radiographs were taken at the end of expiration, by holding the latter position and refraining from swallowing while the film was exposed [31]. These two factors had some influence on the dimensions of the upper airway, but as the present study was based on a large sample size, and we excluded the cephalograms in which a swallowing action was detected, the reference values derived in this study can represent the norms of children in this age group. Thirdly, transversal airway dimension could not be analyzed due to the limitation of two-dimensional lateral cephalometry.

## Conclusions

1. Cephalometric norms for the upper airway of Chinese children have been established providing gender-specific standards. Chinese boys tend to have more risk factors of upper airway for SDB than Chinese girls.

2. Ethnic differences were found in the upper airway dimensions between Chinese and Caucasian children. Chinese children had a greater depth of the retroglossal oropharynx, and Chinese boys had a more anterior and inferior position of the hyoid bone than Caucasian boys.

3. Generally, the associations between the upper airway and craniofacial structures were weak, but the mandibular body length and the craniocervical inclination seemed to have a more prominent correlation with the upper airway than other craniofacial structures.

## Competing interests

The authors declare that they have no financial and non-financial competing interest. This study was funded by the research funding of the University of Hong Kong.

## Authors’ contributions

MG carried out the cephalometric analysis, drafted and finalized the manuscript. CPJM, RWKW, UH and YQY participated the design of the study, interpreted the data and revised the manuscript. All authors read and approved the final manuscript.

## Authors’ information

MG, Clinical Assistant Professor, Department of Paediatric Dentistry and Orthodontics, Faculty of Dentistry, The University of Hong Kong, Hong Kong SAR, China

CPJM, Clinical Professor, Department of Dental Public Health, Faculty of Dentistry, The University of Hong Kong, Hong Kong SAR, China

RWKW, Consultant, Department of Dentistry and Maxillofacial Surgery Cleft Center (Craniofacial Orthodontics), United Christian Hospital, Hong Kong SAR, China

UH, Emeritus Professor, Faculty of Dentistry, The University of Hong Kong, Hong Kong SAR, China

YQY, Clinical Assistant Professor, Department of Paediatric Dentistry and Orthodontics, Faculty of Dentistry, The University of Hong Kong, Hong Kong SAR, China

## References

[B1] Katyal V Pamula Y Martin AJ Daynes CN Kennedy JD Sampson WJ Craniofacial and upper airway morphology in pediatric sleep-disordered breathing: Systematic review and meta-analysis Am J Orthod Dentofacial Orthop 2013 143 1 20 30 e23 10.1016/j.ajodo.2012.08.021 23273357

[B2] Flores-Mir C Korayem M Heo G Witmans M Major MP Major PW Craniofacial morphological characteristics in children with obstructive sleep apnea syndrome: a systematic review and meta-analysis J Am Dent Assoc 2013 144 3 269 277 10.14219/jada.archive.2013.0113 23449902

[B3] Katz ES D’Ambrosio CM Pathophysiology of pediatric obstructive sleep apnea Proc Am Thorac Soc 2008 5 2 253 262 10.1513/pats.200707-111MG 18250219 PMC2645256

[B4] Carroll JL Obstructive sleep-disordered breathing in children: new controversies, new directions Clin Chest Med 2003 24 2 261 282 10.1016/S0272-5231(03)00024-8 12800783

[B5] Marcus CL Brooks LJ Ward SD Draper KA Gozal D Halbower AC Jones J Lehmann C Schechter MS Sheldon S Shiffman RN Spruyt K Diagnosis and management of childhood obstructive sleep apnea syndrome Pediatrics 2012 130 3 e714 e755 10.1542/peds.2012-1672 22926176

[B6] Benninger M Walner D Obstructive sleep-disordered breathing in children Clin Cornerstone 2007 9 Suppl 1 S6 S12 17584620 10.1016/s1098-3597(07)80004-4

[B7] Witmans M Young R Update on pediatric sleep-disordered breathing Pediatr Clin North Am 2011 58 3 571 589 10.1016/j.pcl.2011.03.013 21600343

[B8] Arens R McDonough JM Costarino AT Mahboubi S Tayag-Kier CE Maislin G Schwab RJ Pack AI Magnetic resonance imaging of the upper airway structure of children with obstructive sleep apnea syndrome Am J Respir Crit Care Med 2001 164 4 698 703 10.1164/ajrccm.164.4.2101127 11520739

[B9] Fregosi RF Quan SF Kaemingk KL Morgan WJ Goodwin JL Cabrera R Gmitro A Sleep-disordered breathing, pharyngeal size and soft tissue anatomy in children J Appl Physiol 2003 95 5 2030 2038 12897029 10.1152/japplphysiol.00293.2003

[B10] Isono S Shimada A Utsugi M Konno A Nishino T Comparison of static mechanical properties of the passive pharynx between normal children and children with sleep-disordered breathing Am J Respir Crit Care Med 1998 157 4 Pt 1 1204 1212 9563740 10.1164/ajrccm.157.4.9702042

[B11] Cozza P Polimeni A Ballanti F A modified monobloc for the treatment of obstructive sleep apnoea in paediatric patients Eur J Orthod 2004 26 5 523 530 10.1093/ejo/26.5.523 15536841

[B12] Kawashima S Niikuni N Chia-hung L Takahasi Y Kohno M Nakajima I Akasaka M Sakata H Akashi S Cephalometric comparisons of craniofacial and upper airway structures in young children with obstructive sleep apnea syndrome Ear Nose Throat J 2000 79 7 499 502 505-496 10935301

[B13] Zettergren-Wijk L Forsberg CM Linder-Aronson S Changes in dentofacial morphology after adeno-/tonsillectomy in young children with obstructive sleep apnoea–a 5-year follow-up study Eur J Orthod 2006 28 4 319 326 10.1093/ejo/cji119 16648209

[B14] Deng J Gao X A case–control study of craniofacial features of children with obstructed sleep apnea Sleep Breath 2012 16 4 1219 1227 10.1007/s11325-011-0636-4 22302200

[B15] Pirila-Parkkinen K Pirttiniemi P Nieminen P Tolonen U Pelttari U Lopponen H Dental arch morphology in children with sleep-disordered breathing Eur J Orthod 2009 31 2 160 167 10.1093/ejo/cjn061 19028674

[B16] Zucconi M Caprioglio A Calori G Ferini-Strambi L Oldani A Castronovo C Smirne S Craniofacial modifications in children with habitual snoring and obstructive sleep apnoea: a case-control study Eur Respir J 1999 13 2 411 417 10.1183/09031936.99.13241199 10065690

[B17] Vieira BB Itikawa CE de Almeida LA Sander HS Fernandes RM Anselmo-Lima WT Valera FC Cephalometric evaluation of facial pattern and hyoid bone position in children with obstructive sleep apnea syndrome Int J Pediatr Otorhinolaryngol 2011 75 3 383 386 10.1016/j.ijporl.2010.12.010 21216478

[B18] Vieira BB Itikawa CE de Almeida LA Sander HH Aragon DC Anselmo-Lima WT Matsumoto M Valera FC Facial features and hyoid bone position in preschool children with obstructive sleep apnea syndrome Eur Arch Otorhinolaryngol 2014 271 5 1305 1309 10.1007/s00405-013-2770-z 24162766

[B19] Pirila-Parkkinen K Lopponen H Nieminen P Tolonen U Pirttiniemi P Cephalometric evaluation of children with nocturnal sleep-disordered breathing Eur J Orthod 2010 32 6 662 671 10.1093/ejo/cjp162 20305055

[B20] Major MP Flores-Mir C Major PW Assessment of lateral cephalometric diagnosis of adenoid hypertrophy and posterior upper airway obstruction: a systematic review Am J Orthod Dentofacial Orthop 2006 130 6 700 708 10.1016/j.ajodo.2005.05.050 17169731

[B21] Pirila-Parkkinen K Lopponen H Nieminen P Tolonen U Paakko E Pirttiniemi P Validity of upper airway assessment in children: a clinical, cephalometric, and MRI study Angle Orthod 2011 81 3 433 439 10.2319/063010-362.1 21261486 PMC8923553

[B22] Aboudara C Nielsen I Huang JC Maki K Miller AJ Hatcher D Comparison of airway space with conventional lateral headfilms and 3-dimensional reconstruction from cone-beam computed tomography Am J Orthod Dentofacial Orthop 2009 135 4 468 479 10.1016/j.ajodo.2007.04.043 19361733

[B23] McNamara JA Jr A method of cephalometric evaluation Am J Orthod 1984 86 6 449 469 10.1016/S0002-9416(84)90352-X 6594933

[B24] Samman N Mohammadi H Xia J Cephalometric norms for the upper airway in a healthy Hong Kong Chinese population Hong Kong Med J 2003 9 1 25 30 12547953

[B25] Cooke MS Wei SH Cephalometric standards for the Southern Chinese Eur J Orthod 1988 10 3 264 272 10.1093/ejo/10.3.264 3053214

[B26] Lundstrom A Cooke MS Proportional analysis of the facial profile in natural head position in Caucasian and Chinese children Br J Orthod 1991 18 1 43 49 2025622 10.1179/bjo.18.1.43

[B27] Hou HM Sam K Hagg U Rabie AB Bendeus M Yam LY Ip MS Long-term dentofacial changes in Chinese obstructive sleep apnea patients after treatment with a mandibular advancement device Angle Orthod 2006 76 3 432 440 16637723 10.1043/0003-3219(2006)076[0432:LDCICO]2.0.CO;2

[B28] Dahlberg G Statistical Methods for Medical and Biological Students 1940 London: Allen and Unwin

[B29] Arens R Marcus CL Pathophysiology of upper airway obstruction: a developmental perspective Sleep 2004 27 5 997 1019 15453561 10.1093/sleep/27.5.997

[B30] Coccaro PJ Coccaro PJ Jr Dental development and the pharyngeal lymphoid tissue Otolaryngol Clin North Am 1987 20 2 241 257 3299208

[B31] Battagel JM Johal A Kotecha B A cephalometric comparison of subjects with snoring and obstructive sleep apnoea Eur J Orthod 2000 22 4 353 365 10.1093/ejo/22.4.353 11029825

[B32] Li AM So HK Au CT Ho C Lau J Ng SK Abdullah VJ Fok TF Wing YK Epidemiology of obstructive sleep apnoea syndrome in Chinese children: a two-phase community study Thorax 2010 65 11 991 997 10.1136/thx.2010.134858 20965935

[B33] Ursi WJ Trotman CA McNamara JA Jr Behrents RG Sexual dimorphism in normal craniofacial growth Angle Orthod 1993 63 1 47 56 8507031 10.1043/0003-3219(1993)063<0047:SDINCG>2.0.CO;2

[B34] Sheng CM Lin LH Su Y Tsai HH Developmental changes in pharyngeal airway depth and hyoid bone position from childhood to young adulthood Angle Orthod 2009 79 3 484 490 10.2319/062308-328.1 19413400

[B35] Li H Lu X Shi J Shi H Measurements of normal upper airway assessed by 3-dimensional computed tomography in Chinese children and adolescents Int J Pediatr Otorhinolaryngol 2011 75 10 1240 1246 10.1016/j.ijporl.2011.06.022 21816490

[B36] Taylor M Hans MG Strohl KP Nelson S Broadbent BH Soft tissue growth of the oropharynx Angle Orthod 1996 66 5 393 400 8893109 10.1043/0003-3219(1996)066<0393:STGOTO>2.3.CO;2

[B37] Mislik B Hanggi MP Signorelli L Peltomaki TA Patcas R Pharyngeal airway dimensions: a cephalometric, growth-study-based analysis of physiological variations in children aged 6-17 Eur J Orthod 2014 36 3 331 339 10.1093/ejo/cjt068 24058163

[B38] Wu J Hagg U Rabie AB Chinese norms of McNamara’s cephalometric analysis Angle Orthod 2007 77 1 12 20 10.2319/021606-62R.1 17029538

[B39] Redline S Tishler PV Schluchter M Aylor J Clark K Graham G Risk factors for sleep-disordered breathing in children. Associations with obesity, race, and respiratory problems Am J Respir Crit Care Med 1999 159 5 Pt 1 1527 1532 10228121 10.1164/ajrccm.159.5.9809079

[B40] Khoo SM Tan WC Ng TP Ho CH Risk factors associated with habitual snoring and sleep-disordered breathing in a multi-ethnic Asian population: a population-based study Respir Med 2004 98 6 557 566 10.1016/j.rmed.2003.11.017 15191042

[B41] Ozdemir H Altin R Sogut A Cinar F Mahmutyazicioglu K Kart L Uzun L Davsanci H Gundogdu S Tomac N Craniofacial differences according to AHI scores of children with obstructive sleep apnoea syndrome: cephalometric study in 39 patients Pediatr Radiol 2004 34 5 393 399 10.1007/s00247-004-1168-x 15024528

[B42] Paal P Niederklapfer T Keller C von Goedecke A Luckner G Pehboeck D Mitterlechner T Herff H Riccabona U Wenzel V Head-position angles in children for opening the upper airway Resuscitation 2010 81 6 676 678 10.1016/j.resuscitation.2010.01.022 20346568

[B43] Muto T Takeda S Kanazawa M Yamazaki A Fujiwara Y Mizoguchi I The effect of head posture on the pharyngeal airway space (PAS) Int J Oral Maxillofac Surg 2002 31 6 579 583 10.1054/ijom.2002.0279 12521311

[B44] Ozbek MM Miyamoto K Lowe AA Fleetham JA Natural head posture, upper airway morphology and obstructive sleep apnoea severity in adults Eur J Orthod 1998 20 2 133 143 10.1093/ejo/20.2.133 9633167

